# Rationally designed La and Se co-doped bismuth ferrites with controlled bandgap for visible light photocatalysis

**DOI:** 10.1039/c9ra03064f

**Published:** 2019-05-31

**Authors:** M. Umar, Nasir Mahmood, Saif Ullah Awan, Sabeen Fatima, Asif Mahmood, Syed Rizwan

**Affiliations:** Physics Characterization and Simulations Lab (PCSL), School of Natural Sciences (SNS), National University of Science and Technology (NUST) Islamabad 44000 Pakistan syedrizwanh83@gmail.com +92 51 9085 5599; School of Electrical and Computer Engineering, RMIT University 124 La Trobe Street 3001 Melbourne Victoria Australia; Department of Electrical Engineering, NUST College of Electrical and Mechanical Engineering, National University of Sciences and Technology (NUST) Islamabad 44000 Pakistan; School of Chemical and Biomolecular Engineering, The University of Sydney 2006 Sydney Australia asif.mahmood@sydney.edu.au +61 3 9925 5439

## Abstract

Development of efficient visible light photocatalysts for water purification and hydrogen production by water splitting has been quite challenging. The activities of visible light photocatalysts are generally controlled by the extent of absorption of incident light, band gap, exposure of catalyst surface to incident light and adsorbing species. Here, we have synthesized nanostructured, La and Se co-doped bismuth ferrite (BLFSO) nanosheets using double solvent sol–gel and co-precipitation methods. Structural analysis revealed that the La and Se co-doped BFO *i.e.* Bi_0.92_La_0.08_Fe_1−*x*_Se_*x*_O_3_ (BLFSO) transformed from perovskite rhombohedral to orthorhombic phase. As a result of co-doping and phase transition, a significant decrease in the band gap from 2.04 eV to 1.76 eV was observed for BLFSO-50% (having Se doping of 50%) which requires less energy during transfer of electrons from the valence to the conduction band and ultimately enhances the photocatalytic activity. Moreover, upon increase in Se doping, the BLFSO morphology gradually changed from particles to nanosheets. Among various products, BLFSO-50% exhibited the highest photocatalytic activities under visible light owing to homogenous phase distribution, regular sheet type morphology and larger contact with dye containing solutions. In summary, La, Se co-doping is an effective approach to tune the electronic structure of photocatalysts for visible light photocatalysis.

## Abstract 2

Development of efficient visible light photocatalysts for water purification and hydrogen production by water splitting has been quite challenging.

## Graphical abstract

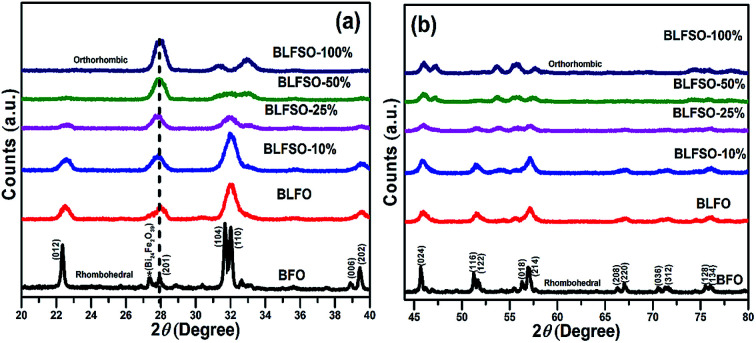

## 1. Introduction

Nanoscale semiconductor-based photocatalysts have elicited significant consideration over the last decades due to their possible applications in solar energy transformation and environmental purification.^1^ Amongst them, titanium dioxide (TiO_2_) has been demonstrated to be a dominant photocatalyst for the degradation of abundant organic amalgams^2^ despite the fact that it can only react to UV light due to its wide bandgap (∼3.2 eV). It is well known that the morphology, band-structures, and nanocomposites with various rare-earth metals could be the reasons for the improving photocatalytic activity of TiO_2_.^3–8^

As solar energy comprises 47% visible and only 2% UV radiation, these photocatalysts have inadequate activity under solar electromagnetic radiation.^11^ To efficiently make use of solar energy that contains mostly visible light, it is critical to discover visible-light-driven photocatalysts.^9^ However, it is quite challenging to design visible light driven photocatalysts because of the large bandgap which limits excitation in the visible region and hence limits the catalytic performance of the designed nanomaterials. Several efforts have been made to design effective visible light driven photocatalysts, however, realization of photocatalysis in visible region is still far from reality.

In recent years, the bismuth ferrite BiFeO_3_ (BFO) has attracted great interest due to its multiferroics properties as this system shows the ferroelectricity and anti-ferromagnetism.^10^ Besides its multiferroics property, current studies also revealed promising visible-light driven photocatalytic properties for BFO.^12,13^ The BFO as photocatalyst has attained a great attention due to its small band gap (2.2 to 2.7 eV), chemical stability, low cost and good response to visible-light irradiation.^14^ Furthermore, magnetic properties of BiFeO_3_ can assist in reprocessing the photocatalyst after the reaction *via* external magnetic field.^14^ This avoids the damage of the catalyst over the rotations and makes the catalyst cost-effective. There by, BiFeO_3_ can act as a noble candidate for visible-light photocatalytic degradation of organic pollutants/dyes.^15^ Still, as talented as BFO material is, a few shortcomings need to overcome prior to tangible commercial and industrial applications. One serious difficulty is the high leakage current in BFO materials, essentially initiating from numerous impurity phases *e.g.* Bi_2_Fe_4_O_9_, Bi_25_FeO_40_ and Bi_36_Fe_24_O_57_ as well as oxygen vacancies, resulting in poor photocatalytic behavior.^16^ In addition, relatively larger bandgap (2.2–2.7) further limits the excitation upon light irradiation. Owing to these shortcomings, the BFO based materials have exhibited very poor photocatalytic degradation efficiencies^14,17–21^ and the work is still going on in this field for improving the catalytic activity of multi ferric systems under visible light. Hence, it is pertinent to find ways to tailor the chemistry of BFO materials so as to obtain pure phase product with lower bandgap in order to harness maximum activities under visible light irradiation.^22–26^

Here, we present a straightforward methodology for tailoring the inherent structure of BFO in such a way that a single phase product could be obtained with much lower bandgap of ∼1.76 eV having sheet type morphology for enhanced contact with incident light and adsorbing species. The product was synthesized by simple wet-chemical and double solvent sol–gel method. Keeping in mind the high probability of recombination between photo-generated electrons and holes in single phase products, we introduced secondary dopants in the form of lanthanum (La) and selenium (Se) to control the recombination and enhance the number of delocalized electrons. A series of experiments were carried out in order to understand the effect of dopant upon morphology, and bandgap and the resulting photocatalytic activity for co-doped (La, Se) BFO nanosheets. Structural changes, morphologies, electronic properties and optical measurement were performed to analyze the recombination rate of charge carriers and elemental composition of the co-doped series. To the best of our knowledge, this is the first study which reports the control of inherent BFO structure *via* La and Se.

## 2. Experimental details

A series of nanoparticles of (La, Se) co-doped BFO with empirical formula Bi_0.92_La_0.08_Fe_1−*x*_Se_*x*_O_3_, *x* = 0, 10%, 25%, 50%, 100% labelled as BFO, BLFO, BLFSO-10%, BLFSO-25%, BLFSO-50%, BLFSO-100% were synthesized by double solvent sol–gel method. These samples are abbreviated as “BLFSO” with varying concentration of Se from 10% to 100%, at fixed 8% La concentration. All chemicals were purchased from Sigma Aldrich commercial grade and used without further modification. The precursors of Bi(NO_3_)_3_·5H_2_O (99.90%, pure) and La(NO_3_)_3_·6H_2_O (99.90%, pure) were stoichiometrically grinded and both were dissolved in ethylene glycol (C_2_H_6_O_2_) and acetic acid (C_2_H_4_O_2_) with continuing stirring for 2 h at room temperature. On the other hand, Fe(NO_3_)_3_·9H_2_O (99.50%, pure) and Se powder (99.50% pure) were dissolved in acetic acid and stirred for 2 h. Both the prepared solutions were again mixed and stirred for 3 h on magnetic stirring. After that, the solution was dried in an oven at 90 °C for 12 h to get a gel and then calcined in furnace at 600 °C for 3 h. For pure BFO, similar preparation methods have been demonstrated earlier.^26^ Structural analysis of the all samples were performed by X-ray Diffractometer (XRD) in the range of 2*θ* = 20–80° using Cu-Kα (*λ* = 0.15418 nm) radiation with a scanning speed of 2 degree per minute. Field emission electron microscopy (FESEM, Hitachi-S5500) was used for study of the microstructures and morphological analysis. The photon source was an Al Kα (1486.6 eV) source operating at 350 W. He–Cd laser of wavelength 325 nm was used as the excitation source during room temperature photoluminescence (PL) measurement. The PL spectrum was observed using a Hitachi luminescence spectrometer (F-4500). Room temperature UV-Vis absorption spectroscopy measurements were carried out using PerkinElmer, Lambda 950 photo spectrometer system.

## 3. Results analysis and discussion

**Fig. 1:**
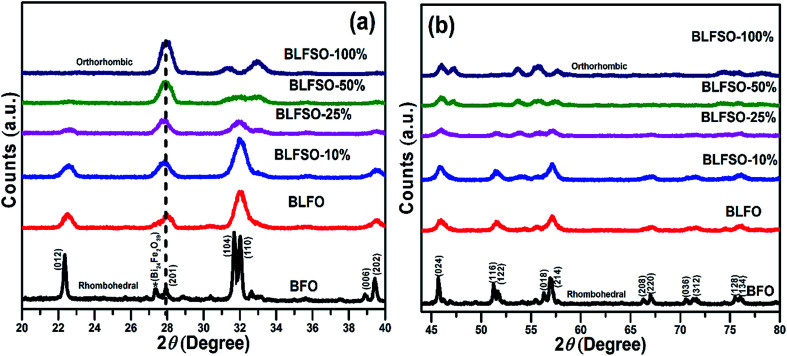
The XRD pattern of BLFSO with varying concentration of Se (a) 20–40 and (b) 42–80 degrees.

The XRD analysis was used to investigate the crystalline structure of BFO and derived nanomaterials and the results are presented in Fig. 1. The XRD patterns of pure BFO (JCPDS card no. 20-0169 and no. 42-0201) corresponds to rhombohedral structure with distorted perovskite structure corresponding to *R*3*c* space group.^27–29^ The dopants were systematically introduced into the BFO crystal lattice. The La was firstly introduced into the crystal lattice and with about 8% addition of La, a phase pure BLFO having a composition of Bi_0.92_La_0.08_FeO_3_ was obtained as revealed by XRD analysis (Fig. 1). After fixing the La doping concentration to 8%, the secondary dopant was introduced into the La doped BFO. Remarkably, (012) peak of pure BFO sample disappeared on increase in the Se concentration from *x* = 0% to *x* = 100% and structural phase transformation from rhombohedral to orthorhombic crystal structure was observed. In addition, a slight shift in the peak positions corresponding to the (104) and (110) towards higher angles were observed with Se co-doping in BLFO nanoparticles which could be ascribed to difference in ionic radius of dopants (Se = 0.64 Å, La = 1.032 Å) than parent ions (Bi^3+^ = 1.03 Å), Fe (Fe^3+^ = 0.78 Å).^30–34^ The XRD analysis revealed that the Se co-doping plays a critical role in phase transformation of pure BFO from rhombohedral to orthorhombic phase. The compositions with relatively lower doping contents (*x* = 0%, 10% and 25%) exhibited mixed phases *i.e.* rhombohedral and orthorhombic. In contrast, compositions with relatively higher doping (*x* = 50% and 100%) exhibited pure orthorhombic phase.

**Fig. 2:**
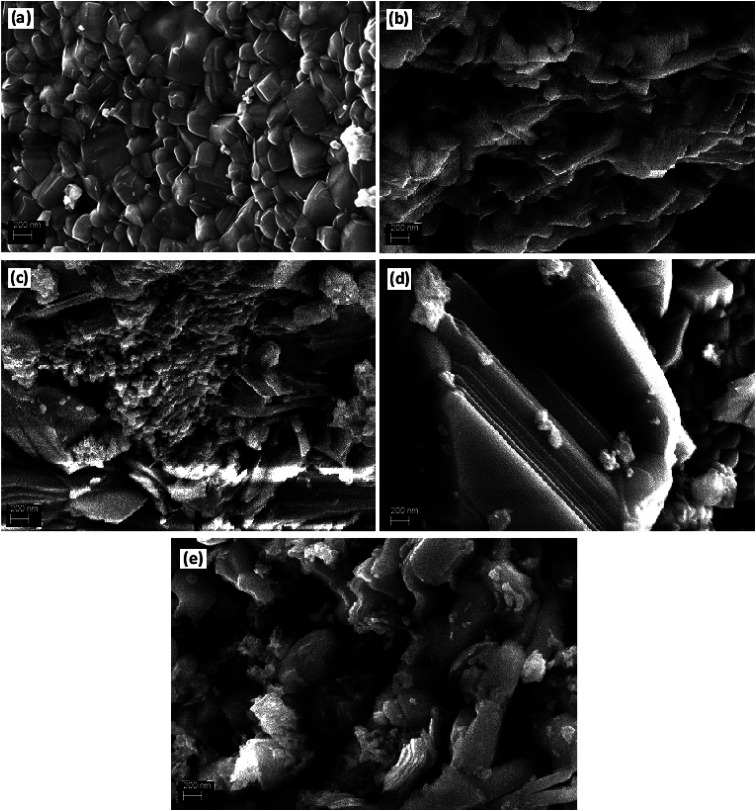
SEM images of (a) BLFO, (b) BLFSO-10% (c) BLFSO-25% (d) BLFSO-50% (e) BLFSO-100%.

To further look into the impact of co-dopant on the morphology of BFO, scanning electron microscope (SEM) measurement were carried out and presented in Fig. 2(a–e). An overall particle type morphology was observed for BLFO as depicted in Fig. 2(a). The doping of Se lead to significant change in particle type morphology of BLFO and resulting Se-doped products exhibit an overall sheet-type morphology. Relatively thin sheets were observed with lower doping of Se (10%) as presented in Fig. 2(b). Subsequent increase in Se concentration lead to relatively larger sheets possibly due to long range 2-dimensional order in the structure as shown in Fig. 2(c). The product obtained with Se doping of ∼50% (Fig. 2(d)) exhibited large and compact sheets which ensure exposure of large surfaces for enhanced catalytic response. Going beyond ∼50% Se doping caused distortions in the sheets leading to irregular structure and hence compromised the overall surface of the product as shown in Fig. 2(e).

**Fig. 3:**
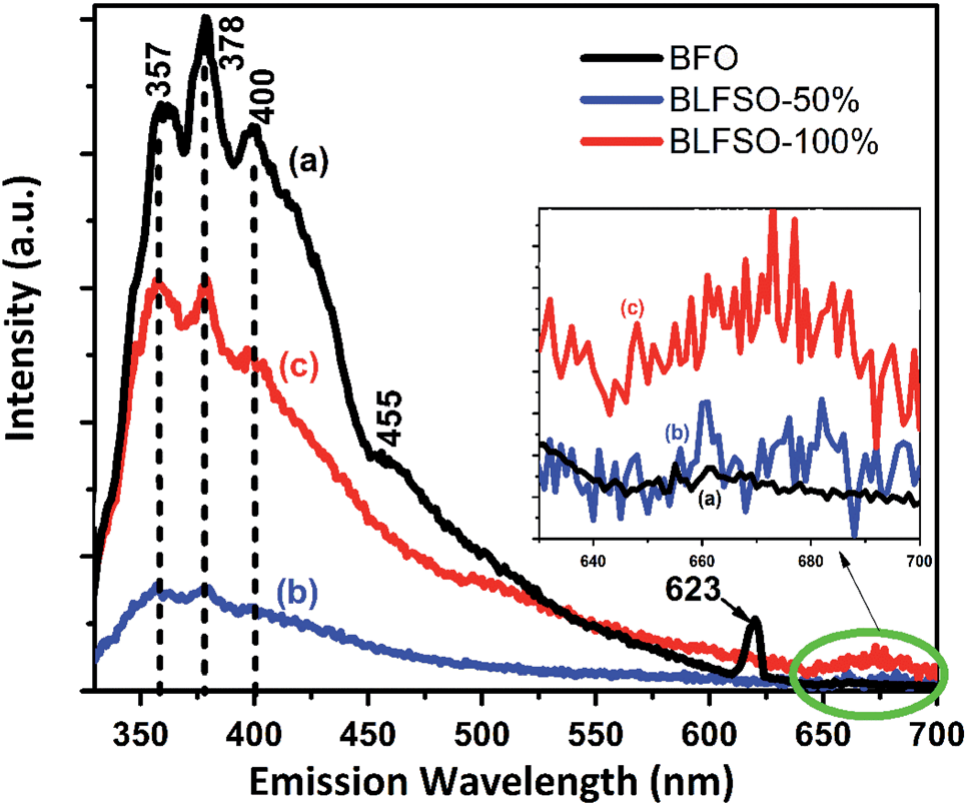
Room temperature PL spectra of nanostructured (a) BFO (b) BLFSO-50% (c) BLFSO-100%.

Room-temperature photoluminescence (PL) emissions at the excitation wavelength of 300 nm were measured to investigate the effect of co-dopants for generation of possible defects within the narrow bandgap of pure BFO system. Modification of band structure in a photocatalyst is fundamentally related to interruption or reduction in the recombination opportunities through the photocatalytic progression.^34–40^Fig. 3 shows the PL spectra of pure and co-doped BFO samples. The PL spectra of each sample has asymmetric broad curve with clear peaks identifications at 357 nm, 378 nm, and 400 nm. The characteristic PL signal at 357 nm is ascribed to the radiative emission of nanostructures during the recombination of electron–hole pairs, while the other signals at 378 nm, 400 nm are due to the emission as a result of the recombination owing to the defects, and donor–acceptor interactions.^41–44^ A small kink corresponding 455 nm was observed in pure BFO which correspond to bandgap of 2.72 eV. In comparison to BFO, the BLFSO-50% exhibited a much shorter bandgap of 2.43 eV at emission wavelength of 510 nm. Interestingly, no impurity was observed close to 520 nm in BFO system as reported previously. However, we noticed an impurity peak only existing in pure BFO nanoparticles at 623 nm. Additionally, broad spectra in the range of 640–700 nm (inset Fig. 3) was observed for BLFSO-50% and BLFSO-100% sample. No such peak at higher wavelength was observed for BFO. It can be speculated that the intense spectrum arise due to the phase transition from rhombohedral to orthorhombic occurred upon increasing Se concentration in the derived products.

Photocatalytic activity is strongly affected by competition between the charge separation and recombination processes and the PL emission spectra have been widely used to estimate the rate of charge recombination.^45,46^ According to the previous studies, the lower the PL emission intensity, the lower is the recombination rate of the photo-generated electron–hole pairs and the greater is the photo-activity of photo-catalyst.^47^ It should be noted that no wavelength shift was observed upon doping of Se in the BFO, rather a strong variation in PL intensity was observed which clearly indicates the recombination behavior in the developed products. For instance, highest intensity of the major signal at 378 nm was observed for BFO which was greatly reduced for co-doped nanostructures particularly for BLFSO-50%. Lower PL intensity for BLFSO-50% nanosheets suggested reduction of radiative emission due to the improved recombination resistance owing to the de-localization of excited electrons in the conduction band. This indicates the possibility of enhanced photocatalytic response, higher stability and durability for the BLFSO-50% nanosheets. In comparison to BLFSO-50%, the PL analysis for BLFO-100% exhibited higher radiative emissions clearly indicating higher recombination rate for higher doping of Se leading to poor catalytic response. It can be assumed that the Se doping of 50% would provide optimal ratio between La^3+^ and Se^4+^ dopants to tailor the intrinsic structure, hence leading to enhanced recombination resistance.

**Fig. 4:**
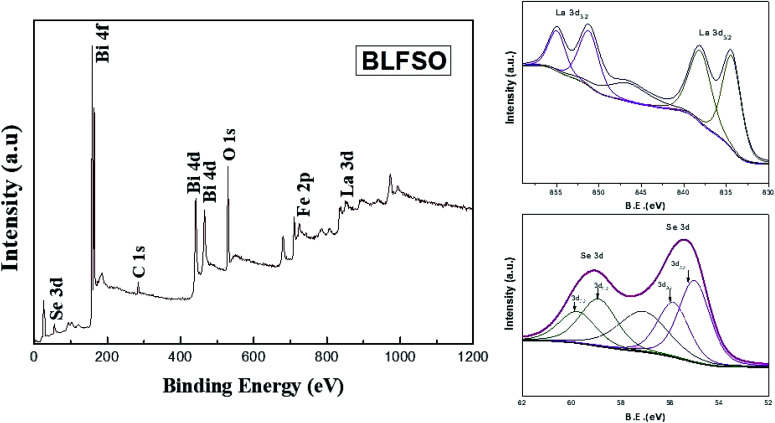
XPS spectra of BLFSO sample.

X-ray photoelectron spectrum has been performed for evaluating the surface chemical states of the BLFSO sample. The peaks corresponding to Bi4f, Bi4d, C1s, O1s, La3d and Se 3d can be shown in Fig. 4. Selenium has two main peaks at 55.4 eV and 59.1 eV corresponding to the oxidation of BFO surface with selenium. Similarly, lanthanum has 834 eV, 838.2 eV, 851.3 eV and 854.9 eV corresponding to La3d3/2 and La3d5/2. Further C1s and O1s peaks are present at 298.8 eV and 545.3 eV. One dominant peak corresponding to Bi4f and two peaks corresponding to Bi4d are clearly visible at binding energy of 168.8 eV, 441 eV and 466 eV. The presence of selenium and lanthanum peaks with a positive shift in the oxygen peak exhibits the bonding of La and Se over the BFO surface which in turns helps in enhancing the charge transfer over the BLFSO surface.^48,49^ The enhancement in charge transfer further reduces the recombination rate in the photocatalytic materials which further helps the photo catalyst in enhancing the dye degradation.^50^

**Fig. 5:**
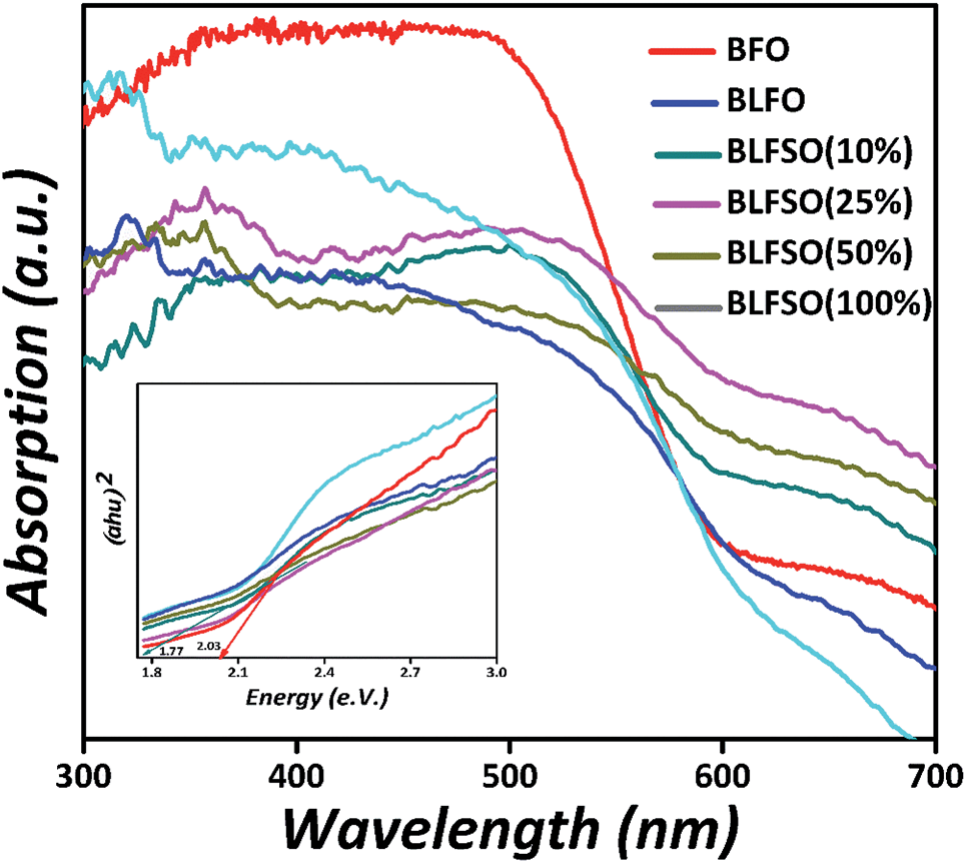
Optical absorption spectra of BLFSO samples.

To investigate the influence of La^3+^ and Se^4+^ co-doping on the optical absorption of BFO, the UV-Vis diffused reflectance spectra (DRS) of the pure BFO and BLFSO samples were measured at room temperature, as shown in Fig. 5. The absorption band edge of the pure BFO nanoparticles appears at 610 nm which is comparable to previous reports^51^ and designates that BFO can react to visible light. In association with pure BFO, the La^3+^ and Se^4+^ co-doped BFO samples displayed an enhanced absorption energy under visible light, and the absorption edge spectra change abruptly to the near infra-red region, which is a pure red shift change in these samples. Thus, the light absorption range is prolonged, which enhances the photocatalytic activity of BLFSO under UV-vis-NIR light treatment. Furthermore, the band-gaps were deliberated using the Kubelka–Munk function^52^ (*αhν*)^2^*versus* photon energy (*hν*) for the direct band-gap semiconductor; the extrapolation of the tail at *y*-axis equals to zero gives the band gap, as shown in the inset of Fig. 5 The band-gaps are 2.03, 1.99, 1.96, 1.80, 1.77, 2.05 eV for the pure BFO, BLFO, BLFSO-10, BLFSO-25, BLFSO-50 and BLFSO-100 samples, respectively. The band-gap of the BLFSO-50 sample is smallest in comparison to that of BFO and other samples. The reduction in bandgap for BLFSO-50 can be ascribed to relatively larger crystals, optimally tailored lattice parameters and defects which all contribute to lowering of the band gap upon introduction of co-dopants.

**Fig. 6:**
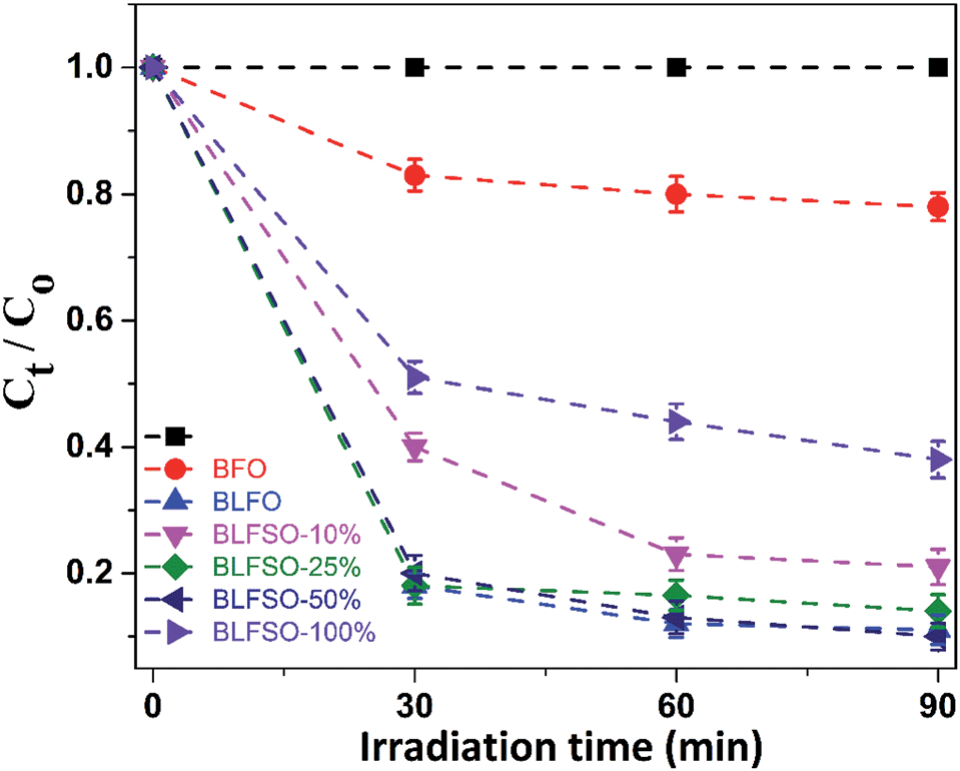
The photo-degradation efficiencies of CR as a function of irradiation time under visible-light for BLFSO series of samples with varying the Se concentration from 10–100%.

**Fig. 7:**
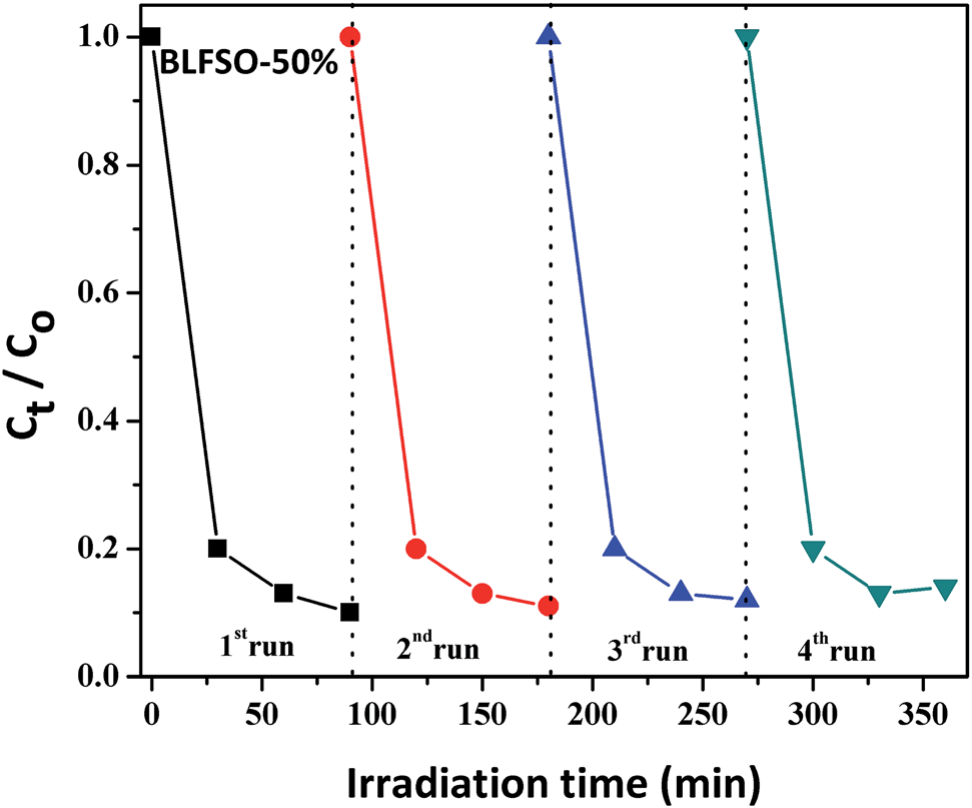
The stability of BLFSO-50% photocatalyst after four cyclic runs under visible light.

The photocatalytic activity of the developed samples was investigated under visible light irradiation using xenon lamp having 300 W power source with 800 nm filters. The Congo Red (CR) was used was used as a model dye in aqueous solution. Fig. 6 represents the photocatalytic activity for various samples as a function of time. The dye solution exhibited limited dye degradation under visible light when no catalyst was used. However, upon addition of designed catalyst, much faster dye degradation was observed probably due to enhanced catalytic reaction on the surface of the catalyst. Highest levels of dye degradation were observed for BLFSO-50% with over 90% dye degradation in first 30 minutes. The degradation level of the BLFSO nanoparticles is higher than the La doped BFO nanofibers^51^ and Se doped BFO.^26^ The dye removal time taken by the BLFSO-50% is very less ass compared to other multi ferric nanoparticle systems (180 min, 270 min, 1 h and).^40,52–54^ In comparison, compositions with other Se doping levels exhibited much poor dye degradation activities. Higher dye degradation activities for BLFSO-50% can be attributed to complete phase transition from rhombohedral to orthorhombic which provide favorable bandgap (∼1.77 eV) and binding energies for enhanced catalysis of dye species. Moreover, homogenous and compact sheet type morphology provide higher active surface area and larger contact with dye solution which eventually enhance the number of exposed sites for catalytic reaction. The activities of compositions with lower Se doping *i.e.* BLFSO-10% and BLFSO-25% were limited by incomplete phase transition which limits the photocatalytic activity. Similarly, the BLFSO-100% exhibited much lower photocatalytic activities (58%) which might be attributed to disruption of sheet type morphology which limits the contact with dye containing solution. Moreover, the stability of a photocatalyst is an important factor for its practical applicability. So, the BLFSO-50% photocatalyst were analyzed for four cyclic runs and as expected it showed approximately the same activity after repeating the photocatalytic process under visible light which confirmed its stability as shown in the Fig. 7.

The mechanism of photocatalytic reaction was investigated to obtain more insights into higher activities obtained from BLFSO samples. Generally, the photocatalysis usually involves three steps: (i) absorption of photons (ii) the production, separation of photo generated e^−^–h^+^ pair (iii) redox reactions. In this process, ·OH radicals act as active specie for degradation of organic compounds such as the CR.^55–61^ These radicals are produced by capturing of their e^−^–h^+^ pair by molecular oxygen and water as described in the following equations:

I Sample + *hν* → sample* (e_CB_^−^ + h_VB_^+^)

II h_VB^+^_ + CR → direct oxidated dye

III h_VB^+^_ + H_2_O → ·OH + H^+^

IV h_VB^+^_ + OH^−^ → ·OH

V e_CB_−__ + O_2_ → ·O_2_^−^

VI ·OH + CR → CO_2_ + H_2_O (by-products)

VII ·O_2_^−^ + CR → CO_2_ + H_2_O (by-products)

These radicals react with organic pollutant (CR in our case) and degrades it into harmless by-products (eqn (VI) and (VII)).^56,62^

## 4. Conclusion

In summary, we report the tailoring of BFO structure with rational addition of dopants to control the inherent crystal structure and obtain much lower bandgap for enhanced visible light photocatalytic response. A series of samples with fixed La concentration and varying concentration of Se (10%, 25%, 50%, 100%) were synthesized *via* simple double solvent sol–gel method and co-precipitation methods. The experimentation revealed that the bandgap of the co-doped samples could be easily tailored by varying the dopant contents and an overall sheet type product could be obtained. The lower bandgap provided easy electron availability upon exposure to incident radiation while the sheet type morphology ensured larger contact between surface of the catalyst and the adsorbing species, thus resulting in enhanced synergistic response and higher catalytic activities. Owing to these attributes, the developed product exhibited excellent photocatalytic activities for model dye, catalyzing more than 90% dye in first 30 minutes of exposure to visible light. We strongly believe that the methodology provide pathway for tailoring inherent structure of ferrite-based catalysts to be used for visible light photocatalysis.

## Author's contribution

M. Umar fabricated and characterized the samples, Saif Ullah Awan contributed in extensive manuscript writing and analysis of results, Nasir Mahmood, helped in characterization, Sabeen Fatima provided help with the XPS measurement and analysis of the sample. Asif Mahmood and Syed Rizwan conceived the research idea and co-supervised/supervised the entire project.

## Conflicts of interest

There are no conflicts to declare.

## Supplementary Material
